# Factors affecting patient-acceptable symptom states and treatment decision in familial Mediterranean fever

**DOI:** 10.55730/1300-0144.5547

**Published:** 2022-06-13

**Authors:** Derya YILDIRIM, İbrahim VASİ, Erdi TAHTA, Rıza Can KARDAŞ, Burcugül ÖZKIZILTAŞ, Hamit KÜÇÜK, Mehmet Akif ÖZTÜRK, Şeminur HAZNEDAROĞLU, Berna GÖKER, Abdurrahman TUFAN

**Affiliations:** 1 Division of Rheumatology, Department of Internal Medicine, Faculty of Medicine, Gazi University, Ankara, Turkey; 2 Department of Internal Medicine, Abdurrahman Yurtaslan Education and Research Hospital, Ankara, Turkey

**Keywords:** Familial Mediterranean fever, patient-acceptable symptom state, treatment, patient participation

## Abstract

**Background/aim:**

Familial Mediterranean fever [FMF] is the most common autoinflammatory disease characterized by inflammatory attacks of fever and polyserositis. Patients’ quality of life is significantly affected due to recurrent excruciating pain attacks and complications. This study is performed to evaluate the parameters most affecting patients’ satisfaction from treatment.

**Materials and methods:**

Three hundred and forty-six consecutive patients diagnosed with FMF were enrolled in this study. Current treatment, acute phase proteins, number, type, and severity of predominant attacks, absenteeism from work/school in the last three months were recorded, and the participants were asked whether they needed additional treatment to evaluate Patient Acceptable Symptom State (PASS) status.

**Results:**

Mean age of the overall group was 38.2 ± 11.7 years (62.4% female, 37.6% male). Two hundred and twenty-seven patients were treated with colchicine, 97 patients with colchicine plus Interleukin-1 (IL-1) antagonist, and 22 only with IL-1 antagonist (67.1%, 26.3%, 6.64% in order). Of the overall group, 33.8% (n = 117) believed to need additional treatment options. Additional treatment need of patients was significantly affected by work impairment due to attacks, absent days from work, disease activity, the discomfort of patients during attacks, the number of attacks, and treatment options; but not by the level of acute-phase proteins between attacks.

**Conclusion:**

PASS score is significantly related to clinical parameters and quality of life. Patients’ PASS scores and treatment choices are notably affected by the severity and frequency of attacks and absenteeism from work/school. Clinical activity and quality of life should be evaluated at every visit to provide patients’ satisfaction with treatment.

## 1. Introduction

FMF is the most common autoinflammatory disorder characterized by recurrent attacks of fever with many clinical conditions related to serositis. Increased risk of secondary amyloidosis, which mainly affects renal and vascular function in untreated or insufficiently treated patients, colchicine resistance-intolerance, and incompliance to follow-up of patients are main obstacles to managing the disease [1–3]. FMF can significantly impair the quality of life due to recurrent attacks, severe pain, and fever during attacks, leading patients to be bedridden during the attack. For this reason, various quality-of-life standard measures are frequently used. Patient global visual analog scale (PG-VAS) scales are not a rational and objective method, but the simplicity and reliability may provide useful opinions about preference-based approaches and quality of life [4]. Autoinflammatory disease activity index (AIDAI) has been recently used for evaluating the frequency and severity of attacks, using a single-format disease-adapted patient diary for hereditary fever syndromes [5]. The Institute of Medicine acknowledges that shared decision-making is a central component to patient-centered care, it is essential to improving quality of care, and it can reduce disparities in clinical outcomes [6]. Sharing treatment decisions with the patient is an important element that ensures regular follow-ups and treatment compliance. No data comprehensively presents the factors affecting the patient’s treatment satisfaction in FMF. We aim to assess factors of clinical and laboratory outcomes and factors affecting patients’ decision-making about treatment in patients with FMF.

## 3. Materials and methods

The study was conducted between March and September 2021 in the Rheumatology Clinic of Gazi University Faculty of Medicine. FMF diagnosis was established according to Eurofever criteria [7]. Demographic features, comorbidities, clinical manifestations, detailed attack characteristics, treatment responses, disease complications, family history, laboratory features, and MEFV mutations were recorded. All patients were prospectively monitored for the frequency, duration, severity of attacks, PG-VAS during attack, AIDAI scores, laboratory 2 parameters, compliance, and adverse effects of therapy and work productivity as absenteeism and presenteeism on each visit with 3-to-6-month intervals. Patient-acceptable symptom state (PASS) is defined as the highest level of symptom beyond which patients consider themselves well. Patients were classified as PASS-positive if they were satisfied with the treatment according to their scores and PASS-negative if they needed additional treatment. According to the treatment, the study group was divided into three divisions: 1. only colchicine, 2. colchicine plus IL-1 antagonists, and 3. IL-1 antagonist. The third group had colchicine intolerance due to various reasons such as diarrhea, elevated liver function test, and cytopenia. A complete response to colchicine was defined as less than one attack per 6 months. Colchicine resistance was defined as one or more attacks per month and elevated acute phase proteins between the attacks [8]. Interleukin 1 inhibitors were used in patients with frequent attacks (≥1 attack per month) and in those with chronic manifestations of disease or amyloidosis plus remarkable inflammatory activity indicated by persistent acute phase elevation despite the maximally tolerated dose of colchicine. In all patients, the initial IL-1 inhibitor was anakinra and switched to canakinumab when adverse effects, inadequate response, allergic reaction, or intolerance were observed with anakinra, which was approved before canakinumab by the regulatory authority for pharmacoeconomic reasons, off-label permission was taken from health authorities when needed. Ethics committee approved the study.

For quality of life assessment, all patients were asked for VAS, AIDAI scores, PASS status, frequency, severity, type and duration of attacks, absent days from work/school in the last 3 months, and additional treatment needs. The original AIDAI diary contains 13 items as follows: (a) fever ≥ 38 °C; (b) overall symptoms; (c) abdominal pain; (d) nausea/vomiting; (e) diarrhea; (f) headaches; (g) chest pain; (h) painful nodes; (i) arthralgia or myalgia; (j) swelling of the joints; (k) eye manifestations; (l) skin rash; (m) pain relief taken. In the original version of the AIDAI, 11 out of 12 items were scored by the patients/parents as 0 = absent, 1 = minor, 2 = mild, 3 = severe, while fever was scored as 0 = absent or 1 = present for a total score in a single day of 0–34 and a month of 31 days of 0–1054 [5]. We also take into account these parameters for the last 1 month and scores between 0 and 372.

All statistical analyses were performed with SPSS v. 17.0 software (SPSS Inc., Chicago, IL, USA). Descriptive values were presented by mean (standard deviation, SD) and median (minimum-maximum), and categorical variables as percentages. The numeric variables were investigated using visual (histograms and probability plots) and analytical (the Kolmogorov–Smirnov test) methods to determine the distribution of data. Because all numeric parameters showed abnormal distribution, the Mann–Whitney U and Kruskal–Wallis tests were used according to the number of study groups. The chi-squared test is applied for categorical variables. Post hoc analysis of categorical variables was also applied with chi-squared test Bonferroni correction. p-values less than 0.05 was accepted as statistically significant.

## 3. Results

A total of three hundred and forty-six consecutive patients diagnosed with FMF were included in this study (216 female, 130 male, mean age 38.2 ± 11.7 years). Patients were divided into three treatment groups: 1. only colchicine, 2. combination of colchicine and IL-1 antagonists, and 3. IL-1 antagonists alone. Treatment groups were compared about PASS status, AIDAI, patient global VAS scores, absenteeism from work/school, type-duration-severity of attacks, and acute-phase proteins.

Table 1 shows the differences between the PASS-positive and PASS-negative groups. There was no significant difference in sex between PASS-positive and -negative patients (p = 0.218).

PASS status of patients was also significantly affected by treatment options (p = 0.025). Seventy-five percent of PASS-positive patients were using colchicine alone, 21.3% were using colchicine together with IL-1 antagonist, and 2.8% were using only IL-1 antagonist. Patients using only colchicine plus IL-1 antagonists were more satisfied with treatment when compared to other treatment options (p = 0.01 for both). There was no significant difference in PASS status between only-colchicine and only IL-1–antagonist treatment groups. Absenteeism due to FMF attacks was significantly associated with PASS status. Of the overall group, 29.7% complained about work/school impairment and absenteeism because of attacks. 18.4% of patients without workday loss and 52% of those with workday loss expressed additional treatment need, which was statistically significant (p < 0.05). AIDAI and VAS scores were also significantly associated with the PASS score. PASS-negative patients had significantly higher global VAS and AIDAI scores (p < 0.05 for both). Interattack C-reactive protein (CRP) and sedimentation levels were not associated with PASS status (p = 0.413 and p = 0.671, in order).

AIDAI, PG-VAS, and proteinuria were significantly different between all study groups (p < 0.001, p = 0. 006, p = 0.001). CRP and sedimentation levels were not different (p = 0.743, p = 0.408 in order; Table 2). Subgroup analyses with the Mann–Whitney U test showed significantly higher VAS and AIDAI scores for the patient group treated with colchicine when compared to colchicine plus anti-IL-1 treatment group (p = 0.005 and p = 0.002). Patients treated with only IL-1 antagonist showed significantly lower AIDAI scores than the only-colchicine group and colchicine plus anti-IL-1 treatment groups (p < 0.001, p = 0.001).

## 4. Discussion

The goals of treatment in FMF are improving quality of life (QoL); reducing the frequency, severity, and duration of attacks; and preventing long-term damage, particularly AA amyloidosis by minimizing chronic/subclinical inflammation. Since the disease is usually diagnosed at an early age, the long duration of the disease causes difficulty for close follow-up of patients. Considering the quality of life and preferences of patients may facilitate adherence to treatment.

Colchicine is an anchor drug for achieving remission and avoiding damage [9–11]. Although colchicine is so effective, it still cannot be used in a significant number of patients due to intolerance and resistance problems [12]. In our study group, twenty-three patients were using only IL-1 antagonists because of colchicine intolerance. Major side effects of colchicine are diarrhea, elevation in transaminases, and leukopenia. Colchicine intolerance constitutes a major problem, with almost one-fifth of patients unable to maintain optimal doses which are described as more than 1.5 mg/day [13] Defining colchicine resistance has always been challenging. Ozen et al. described a new definition the patients who have ongoing disease activity while those taking the maximum tolerated dose of colchicine are referred to as colchicine-resistant [8]. In our study, all patients treated with IL-1 antagonist had colchicine resistance. Colchicine could not be given to some of them [n = 22] besides IL-1 antagonists due to intolerance.

IL-1 antagonists are a promising choice of treatment for colchicine-intolerant or -resistant patients [14]. All of the IL-1 antagonists have proven to be an effective choice for reducing the number of attacks and improving quality of life with insignificant side effects [15, 16]. Although clinical experience about the efficacy of IL-1 antagonists is approved, this opinion is based on small-scale data [17,18]. For this reason, it has been deemed appropriate only to be used in addition to colchicine until further studies are conducted [11]. According to our data, patients treated with IL-1 antagonists showed lower VAS and AIDAI scores when compared to the only-colchicine group. This data also supports the previous report which evaluated attack frequency and VAS scores in patients with FMF and treated with IL-1 antagonists [15]. Proteinuria was significantly higher in IL-1–treated groups. This difference may have arisen because the group to which an IL-1 antagonist was administered was usually colchicine-resistant patients whose proteinuria was markedly increased. Moreover, interattack CRP, sedimentation levels, and VAS scores did not differ significantly between the two groups using IL-1 antagonists. From here, it can be discussed whether additional colchicine treatment is required in all patients treated with IL-1 antagonists. Elevation of acute-phase proteins between attacks did not show a significant difference between the treatment groups in our study. Since most patients in the treatment groups were followed closely in the outpatient clinic, none of them had higher acute phase proteins between attacks. Increased acute phase elevation between attacks is positioned as an indication for ongoing inflammation and colchicine unresponsiveness in previous reports 4 [19]. From this, we can conclude that close follow-up of the patient is important in the management of treatment [3]. Patient involvement in the process of medication decision-making can enhance patient satisfaction, understanding, and confidence in decision-making [20, 21]. Patient-reported outcome measures have been used for the assessment of the quality of life in patients with chronic disabling diseases [22]. PASS has been shown as a practical test for evaluating disease activity [23, 24]. PASS status of patients was significantly related to VAS, AIDAI scores, work/study day loss, and treatment options. Considering these data, we can postulate that the factors that are effective in the decision of the patients are the frequency of attacks and the limitation of work.

Working ability and sustaining employment are important determining factors for patients’ therapeutic decisions [25]. Studies revealed compromised work productivity in many diseases including rheumatoid arthritis, spondyloarthritis, systemic lupus erythematosus [26–28]. FMF was also studied and experiencing serositis attacks and severity of pain during attacks was related to work impairment in FMF patients [29]. According to our data, patients’ satisfaction with treatment and PASS scores were related to work impairment. In addition, patients with work impairment had higher AIDAI and VAS scores. Reducing the frequency and severity of the patient’s attacks can provide preventing absenteeism and thus control ongoing inflammation by increasing treatment compliance. Many widely used quality of life scales were applied and reported for patients with FMF like Short Form 36 (SF-36), the World Health Organization Quality of Life Scale Short Form (WHOQOL-BREF), and the Health Assessment Questionnaire (HAQ) [30–32]. Moreover, a specific scale is also available for FMF patients, and it showed strong correlation of clinical status in patients with FMF [33]. Nonetheless, SF-36 was significantly correlated with severity of disease in patients with FMF and treated with IL-1 blockers. Bodur et al. revealed that FMF-QoL was significantly correlated with SF-36 and HAQ and also had a relationship with severity of FMF attacks [34]. Although quality of life scales were not evaluated in our study, it can be postulated that PASS status of patients is also an important predictive parameter about quality of life. The limitations of our study can be expressed as the low number of patients and the inability to document the day of work impairment numerically. Larger-scale studies to be conducted in this area may reveal the factors associated with absenteeism in FMF more clearly. Our study is original as it is the first study to evaluate PASS status and work/school day loss according to treatment groups.

## Figures and Tables

**Table 1:** Comparison of clinical and laboratory conditions between PASS-positive and -negative patients.

	PASS-positive	PASS-negative	p-value

Number of patients	229	117	NA

Age (median, years)	36.5	34	0.141

Sex (female/male)%	65.7%/34.3%	55.3%/44.7%	0.218

Genetic mutation (number of patients)			
M694V homozygosity	42	12	
M694V (if any allele)	104	64	NS
M680I (if any allele)	46	26	
V726A(if any allele)	26	14	
Other mutations	11	1	

AIDAI score (mean)	2	4	**<0.005**

Patient global VAS (mean)	4	6	**<0.005**

Work/study day loss	23.1%	40.6%	**<0.005**

Treatment(colc/colc plus IL1A/only IL1A)	75.9%/21.3%/2.8%	55.3%/42.6%/2.1%	**0.025**

Proteinuria (mg/day)	149.57	317.86	0.932

CRP between attacks (mg/L)	4	4	0.413

Sedimentation between attacks (mm/h)	16	17.5	0.671

Attack types (number of patients)			
Peritonitis	86	46	
Pleuritis	43	21	NS
Fever (only)	13	6	
Arthritis	62	42	
Other	25	8	

AIDAI: autoinflammatory disease activity index, VAS: visual analog scale, colc: colchicine, IL1A: interleukin 1 antagonist, CRP: C reactive protein.

**Table 2:** Comparison of descriptive parameters between the treatment groups.

	Only colchicine	Colchicine plus IL1A	Only IL1A	p-value
Age (median, years)	37	34	38	0.15
Number of patients	227	97	22	NA
Sex (female/male)	66.4%/33.6%	56%/44%	47.8%/52.2%	0.074
Dominant attack	Peritonitis	Fever	Peritonitis	0.847
AIDAI (mean)	3	2	0	0.005
VAS (mean)	5	3	3	0.008
CRP (mg/L)	4	4w	5	0.722
Sedimentation(mm/h)	15	18	13.5	0.145
Proteinuria (mg/day)	71.4	324.8	812.14	0.016
Creatinine (mg/dL)	0.6	0.57	0.82	NS
Percentage of patients with amyloidosis (%)	26.4%	89.6%	100	NA

AIDAI: Autoinflammatory disease activity index, VAS: visual analog scale, colc: colchicine, IL1A: interleukin 1 antagonist, CRP: C reactive protein.
